# Case report: First report on human infection by tick-borne *Babesia bigemina* in the Amazon region of Ecuador

**DOI:** 10.3389/fpubh.2023.1079042

**Published:** 2023-08-04

**Authors:** Manuel Calvopiña, María Montesdeoca-Andrade, Carlos Bastidas-Caldes, Sandra Enriquez, Richar Rodríguez-Hidalgo, Dayana Aguilar-Rodríguez, Philip Cooper

**Affiliations:** ^1^One Health Research Group, Facultad de Medicina, Universidad de las Américas (UDLA), Quito, Ecuador; ^2^Departamento de Hemodiálisis, Hospital de Especialidades Carlos Andrade Marín, Quito, Ecuador; ^3^One Health Research Group, Facultad de Ingeniería y Ciencias Aplicadas (FICA), Universidad de las Américas (UDLA), Quito, Ecuador; ^4^Programa de Doctorado en Salud Pública y Animal, Universidad de Extremadura, Cáceres, Extremadura, Spain; ^5^Instituto de Investigación en Zoonosis (CIZ), Universidad Central del Ecuador, Quito, Ecuador; ^6^Facultad de Medicina Veterinaria y Zootecnia, Universidad Central del Ecuador, Quito, Ecuador; ^7^Escuela de Medicina, Universidad Internacional del Ecuador, Quito, Ecuador; ^8^Institute of Infection and Immunity, St. George's University of London, London, United Kingdom

**Keywords:** babesiosis, *Babesia bigemina*, case report, zoonosis, tick-borne, Ecuador, Amazon

## Abstract

Babesiosis is a protozoan disease acquired by the bite of different species of ticks. More than 100 *Babesia* spp. infect wild and domestic animals worldwide, but only a few have been documented to infect humans. Generally, babesiosis is asymptomatic in immunocompetent persons; however, in immunocompromised can be life-threatening. A 13-year-old boy from the Amazon region presented with a 3-month evolution of fever, chills, general malaise, and arthralgia accompanied by anemia and jaundice. In the last 4 years was diagnosed with chronic kidney failure. By nested-PCR using 18S RNA ribosomal gene as target and DNA sequencing, the phylogenetic analysis showed *Babesia bigemina* as the causative agent in the blood. Treatment with oral quinine plus clindamycin for six continuous weeks was effective with no relapse occurring during 12 months of follow-up. This is the second human case in Ecuador but the first caused by the zoonotic *B. bigemina* which confirms the existence of active transmission that should alert public health decision-making authorities on the emergence of this zoonosis and the need for research to determine strategies to reduce tick exposure.

## Introduction

Babesiosis is an emerging tick-borne zoonosis with a worldwide distribution. The number of reported human cases in the literature has increased over recent years, indicating an extension of risk areas (1). Human infections are caused by some species of *Babesia*, that include but are not limited to *B. microti*, *B. divergens*, *B. duncani*, *B. motasi*, *B. crassa*, and two *Babesia* strains, i.e., *Babesia* spp. KO1 and *Babesia* spp. CN1; the latter two may represent a new *Babesia* species (1, 2). The predominant species causing human infections in the North America is *B. microti*, in Europe it is *B. divergens*, while in Asia several cases with *B. microti, B. venatorum*, and *B. crassa*-like have been reported (3). Other species, such as *B. microti* Kobe-type, *B. microti*-like, and some *B. divergens*–like parasites, have also been implicated in human infections (1, 2, 4). There are rare cases where *Babesia* spp. that normally infect cattle and other animals, cause disease in humans especially if they are asplenic or immunocompromised (5).

Few human cases have been diagnosed from South America. There were two from Brazil without molecular identification of the *Babesia* spp. (6). In Colombia in 2003, the first parasitological confirmed case was reported by *B. bovis*, along with three serological positive cases for *B. bovis* and one for *B. bigemina* (7). In a subsequent study, using PCR, four asymptomatic cases due to *B. bovis* and two of *B. bigemina* were detected (8). However, both reports have been disputed given a lack of more definitive evidence from DNA sequencing (9). In a study in Bolivia, 3.3% of persons were positive for *B. microti* by microscopy and PCR, while seroprevalence was 45.7% (2). A traveler from Uruguay to Spain presented mild symptoms due to *B. microti* infection (10). The only symptomatic case reported to have originated in Ecuador and diagnosed in USA was caused by *B. microti* (11).

Ecuador, located in the northwest of South America, is crossed by the Andes belt that divide into three ecoregions: the Andean temperate region, the Pacific Coast tropical region, and the interior tropical Amazon basin. Non-continental Ecuador includes the Galápagos Islands at 1,369 km from the Pacific coast. Babesiosis in cattle is considered endemic and is a national veterinary public health problem where the main tick vector is the *Rhipicephalus microplus*, a species widely distributed in the tropics, subtropics and Andes, between 0 and 2,600 m altitude (12–14). Furthermore, *Rh. microplus* collected from tropical regions resulted positive for *B. bovis*, *B. bigemina* and co-infections (12, 13). At least 41 Ixodid tick species, 32 species of hard ticks (Ixodidae) and 9 species of soft ticks (Argasidae), belonging to Amblyomma, Dermacentor, Haemaphysalis, Ixodes, and Rhipicephalus genera have been documented in Ecuador, including Galapagos Islands (15, 16), with additional species recorded in the Andes region (17).

The clinical manifestations of babesiosis in immunocompetent persons range from asymptomatic to a mild illness. In contrast, severe illness requiring hospital admission is common in persons who are immunosuppressed or splenectomised; or who have cancer, human immunodeficiency virus infection, haemoglobinopathy, or chronic heart, lung, kidney, or liver disease. The severity of babesiosis depends primarily on the immune status of the patient and may cause death (18). With an infection by *B. divergens, B. duncani* and *B. venatorum*, there seems to be a higher probability of severe disease (19). After a gradual onset of malaise and fatigue, the most common clinical sign is fever, sometimes as high as 40.9°C (105.6°F), with chills and sweats accompanied by headache, myalgia, anorexia, arthralgia, and nausea. In severe infections, fever may be accompanied by splenomegaly, hepatomegaly, jaundice, and acute respiratory failure (18). Babesiosis is a protozoal infection like malaria, confusing both clinical and laboratory diagnosis, and more so in areas where the two diseases are overlapping (11).

The diagnosis of babesiosis is based on epidemiology and clinical presentation (4). Laboratory findings that are consistent with hemolytic anemia include a low hematocrit, hemoglobin, and haptoglobin levels, but elevated reticulocyte count and lactate dehydrogenase level; thrombocytopenia is commonly observed (18). Because of difficulties in the laboratory identification of *Babesia* species that have a similar morphology and because of antigenic cross-reactivity, molecular techniques such as PCR and DNA sequencing are often required for species identification (1). DNA sequencing for the phylogenetic analysis of *Babesia* provides data on nucleotide sequences present in an amplified target gene sequence while PCR generates a large number of copies of a specific DNA fragment, represented as a single band (20). PCR is more sensitive and specific than blood smear (19). Among DNA-based assays, nested-PCR based on 18S RNA small subunit fragment for *Babesia* species have been used extensively for the diagnosis of babesiosis, and is highly sensitive, even at low levels of parasitemia (21).

Patients with mild to moderate babesiosis are treated with combination therapy of atovaquone and azithromycin, and severe cases with clindamycin plus quinine. Based on multiple case reports, a 7-to-10-day course of clindamycin-quinine combination is often used to treat severe babesiosis (3). A minimum of 6 weeks for highly immunocompromised patients is recommended (21).

Here we report a severe case of babesiosis caused by *B. bigemina* in an immunocompromised Ecuadorian child from the Amazon basin region who was treated successfully with the combination of clindamycin plus quinine.

## Case report

A 13-year-old boy, Amerindian of the Shuar ethnic group, was born and raised on a farm located in Guayusa (Lat = −0.24837, Long = −77.06041), province of Orellana, northern part of the Ecuadorian Amazon, 8 hours from Quito, the capital of Ecuador. An important antecedent was that he had suffered from stage 3 chronic kidney disease for the previous 4 years (glomerular filtration rate of 49 mL/min), secondary to an anatomical malformation of the urinary tract. He had never received a blood transfusion. Clinical history revealed that for 3 months the child had presented a clinical picture characterized by a fever of 38 to 39.4°C, accompanied by chills, sweating, anorexia, general malaise, and arthralgia. One month prior to hospital admission, the fever was accompanied by jaundice, initially affecting the sclera and later became generalized, with pain of moderate intensity in the right flank. The child had always resided in the Amazon region where the presence of numerous wild animals including deer (*Mazama americana*), rodents, and insects such as mosquitoes, sandflies, and ticks are abundant. He was first medically managed with antipyretics and antibiotics in a local health center and provincial hospital. Since the fever did not subside and the clinical picture of kidney failure worsened, he was transferred to a referral hospital in Quito. Upon admission, he had a fever of 38°C, heart rate of 114 beats/min, respiratory rate of 22/min, oxygen saturation of 97%, blood pressure 129/65 mm Hg, weight 35.4 Kg, height 136.5 cm, abdominal pain, arthralgia, myalgia, and urinary incontinence with dark urine. On physical examination, pale skin and mucous membranes were observed, as were multiple scratching excoriations on the lower extremities.

The blood tests revealed a white blood cell count of 19,500 mm3 with 67% neutrophils, 19% lymphocytes, 11% eosinophils, 2% basophils, and 1% monocytes. Hemoglobin 6.3 mg/dL, hematocrit 19.7%, platelets 139,000 mm^3^, urea 145 mg/dL (12–54 mg/dL), creatinine 3.5 mg/dL (0.7 to 1.2 mg/dL for men), blood urea nitrogen (BUN) 29 mg/dL (7–20 mg/dL), alanine-aminotransferase 64 U/L (4 to 36 U/L) and aspartate aminotransferase 61 U/L (8 to 33 U/L). The direct Coombs test was positive, and lactate dehydrogenase (LDH) was 295 U/L (140 to 280 U/L). Serological studies for human immunodeficiency virus (HIV), viral hepatitis and immunological tests for malaria, Chagas, leptospirosis, and Lyme disease were all negative. Peripheral thick and thin blood smears for *Plasmodium* spp. and *Trypanosoma cruzi* were negative. No intraerythrocytic structures compatible with *Babesia* spp. were observed.

In imaging studies with sonography, hydroureteronephrosis of the right kidney was observed, while the left one was decreased in size with a loss of corticomedullary relationship and ectasia. The right kidney showed the presence of two ureters that ended together ipsilaterally. A bilobed bladder separated by a septum with a paravesical diverticulum was also observed.

**Figure 1:**
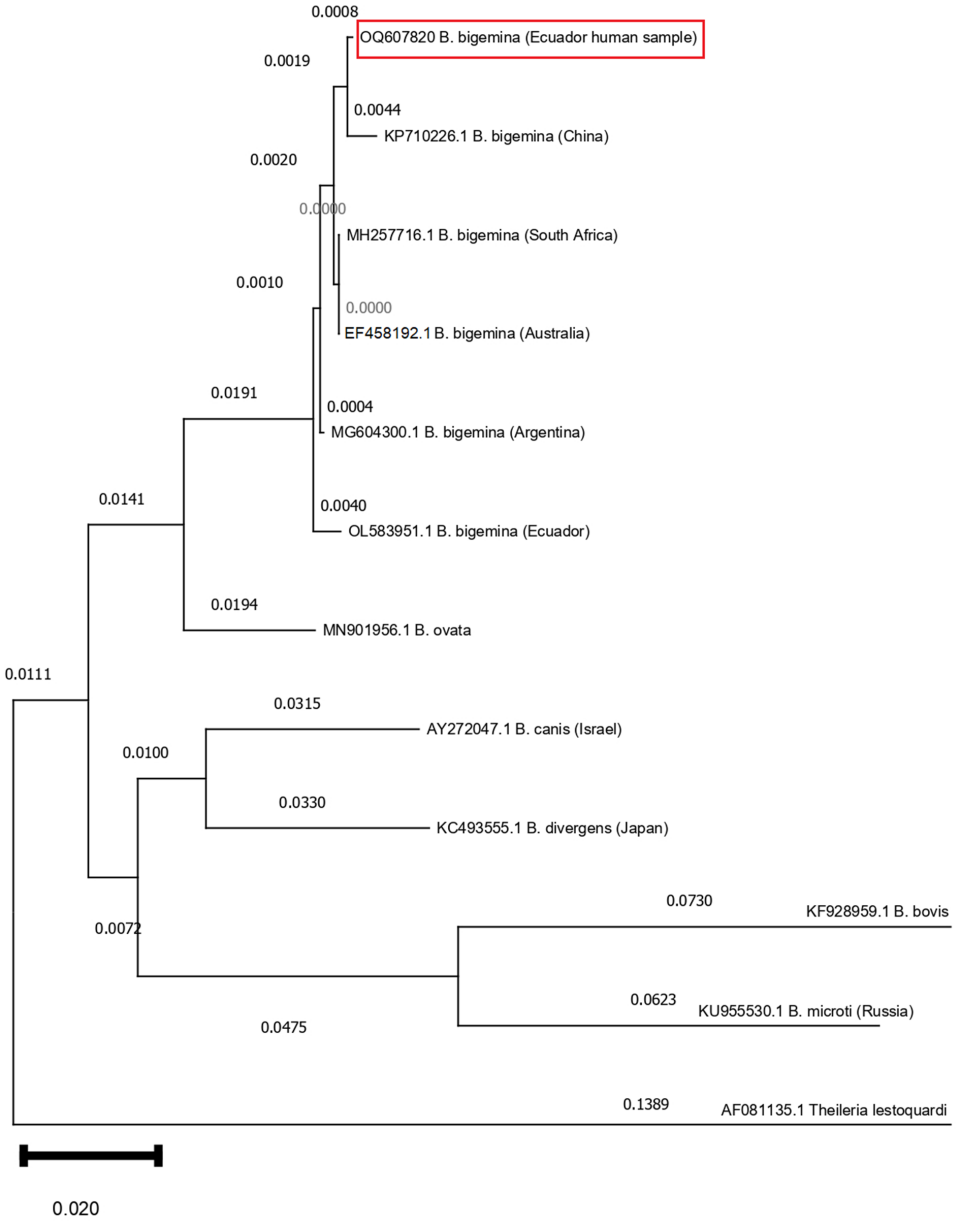
Phylogenetic tree based on the 1,670 nucleotides of the 18S rRNA gene of *Babesia bigemina*. The optimal tree was inferred using the Neighbor-Joining method. The confidence probability (multiplied by 500) that the interior branch length is greater than 0, as estimated using the bootstrap test (1,000 replicates is shown next to the branches). The evolutionary distances were computed using the Tamura Nei model (distribution of 48 parameters). Patient (Ecuador human sample, GenBank accession number OQ607820) showed identities of 99.35% when compared with published *B. bigemina* sequences (KP710226.1, MH257716.1, EF458192.1, MG604300.1, OL583951.1). *Theileria lestoquardi* (AF081135.1) compared as outgroup.

Genomic DNA of whole-blood samples was isolated using commercial Qiagen DNA™-blood MiniPrep kit. A nested PCR for the long fragment of 18S rRNA gene for detection of *Babesia* species were performed. The first PCR step was amplified using the primer set PiroF (5’-GCCAGTAGTC ATATGCTTGTGTTA-3′) and Piro6R (5’-CTCCTTCCTYTAAG TGATAAGGTTCAC-3′). Another pair of primers, Piro1F (5′- CCATGCAGTTCTWAGTAYAARCTTTTA-3′) and Piro5.5R (5’-CCTYTAAGTGATAAGGTTCACAAAACTT-3′) were used in the second PCR (22). In a 2% agarose gel, a clear band of approximately 1,670 bp was observed. The amplicon was sequenced using the Sanger sequencing by an external provider (Macrogen-South Korea). The chromatogram sequence of the gene, obtained with forward and reverse primers, were assembled and consensus sequence was edited using MEGA XI software. The Nucleotide Blast, for the final sequence of 1,546 bp, show a query coverage of 100% and identity of 99.87% for *B. bigemina* using the NCBI resources and a GenBank accession number was obtained (OQ607820.1). The sequences were aligned and compared using the MEGA XI. A phylogenetic tree was constructed through Neighbor Joining method with a Bootstrap of 500 replicates, comparing eleven NCBI sequences from GenBank (Figure 1). Purified DNA of *B. bovis* and *B. bigemina*, kindly supplied by Instituto de Genética de la Universidad Nacional de Colombia, were used as positive controls.

After 32 days of clinical management in the nephrology unit, BUN and creatinine values decreased to 118 mg/dL and 2.2 mg/dL, respectively. Whereas hemoglobin and hematocrit rose to 12.8 mg/dL and 38.8%, respectively. The leukocyte count decreased to 13,400 mm^3^. Anti-*Babesia* treatment was started with oral quinine 300 mg every 8 hours plus clindamycin 300 mg every 12 h, which was continued as an outpatient treatment for 6 continuous weeks. No adverse effects were reported to any of the drugs. Symptoms subsided after 7 days of treatment. No symptomatology was reported at 3-, 6- and 12-month controls and no parasitic forms compatible with *Babesia* were observed in peripheral blood smears. His mother signed the consent for the publication of the case.

## Discussion

Reports of symptomatic cases of human babesiosis worldwide are rare, although in recent years the increased numbers have led experts to consider it as an emerging disease, especially in tropical and subtropical regions (1). The report of this case with severe symptoms in an immunosuppressed boy with the identification of the *Babesia* specie is important in documenting the geographic distribution of human disease in South America and particularly in Ecuador. This is the second symptomatic case in Ecuador, but the first in the Amazon region with *B. bigemina* identified as the causative agent using molecular methods (e.g., n-PCR and DNA sequencing). The first case, which came from the tropical Pacific coastal region and diagnosed in the USA, was caused by *B. microti* (11). This shows that human *Babesia* infection is present in the two tropical ecoregions of the country.

Most human infections are caused by *B. microti* and *B. divergens* and rarely by other species (1, 2, 4, 9). Few human cases by *B. bigemina* have been reported from Colombia and Ecuador in asymptomatic persons residing in the Amazon (7, 8). However, these cases were not confirmed by DNA sequencing: the use of PCR and or serology cannot confirm the *Babesia* species with certainty (1, 9, 20, 23). *Babesia bigemina* is widely distributed geographically and, together with *B. bovis*, is highly infective in livestock (12, 14). In Ecuador, *B. bigemina* has been reported to infect cattle and ticks from the tropical regions, as well as the Andean region (12, 13). Therefore, it is imperative to consider *B. bigemina* as a potential infectious agent which causes severe disease in immunosuppressed patients, as occurred in the present case. We believe that there is underdiagnosis of human babesiosis in Ecuador due to the lack of information among physicians and laboratory technicians, unavailability of sensitive diagnostic tests, and lack of epidemiological studies. Furthermore, there could be a misinterpretation of intra-erythrocytic microorganisms on blood smear with *Plasmodium* spp. (11) since malaria is endemic in Ecuador (24). There is a report of other febrile illnesses transmitted by ticks in the Amazon such as *Rickettsia* (25). Therefore, the presence of *Babesia* infections should be investigated in febrile patients.

The *Babesia* species that infect cattle and livestock in South America are *B. bigemina* and *B. bovis* (2, 26). Using PCR targeting the 18S ribosomal gene in cattle from the Coast and Andes identified both *B. bigemina* and *B. bovis* (12). This information indicates that active transmission in domestic animals is occurring with a permanent risk of infection to humans. The main vectors of *B. bigemina* and *B. bovis* in cattle are ticks of the genus *Rhipicephalus*, *Rh. sanguineus* but mainly *Rh. microplus*, species widely distributed in the tropics and subtropics of South America (13, 26). Although few studies on ticks exist in the country, several tick species of different genera that could act as potential vectors of *B. bigemina* and others, have been documented (12, 14, 15, 17).

The severe clinical symptoms of the present case are a consequence of the child being immunosuppressed due to his chronic kidney failure and delay in the diagnosis (18, 19, 21). In the hematological profile, the observed decrease in hemoglobin and hematocrit was most probably due to the hemolytic anemia. Associated laboratory findings, such as thrombocytopenia, elevation of liver and kidney enzyme levels, jaundice, and dark urine a month before hospitalization, are consistent with a *Babesia* infection confirmed later by nested-PCR and DNA sequencing. In addition, the history of exposure to ticks also supports the diagnosis. No intraerythrocytic forms of *Babesia* spp. were observed, probably due to it being a chronic infection with 3 months of evolution. In chronic cases, parasites may be undetectable by microscopy, so it is important to use the molecular-based techniques, which detects a specific sequence of the nucleic acids of the parasite (20, 23). Along with the progress in molecular techniques, the knowledge of *Babesia* is further expanding and more species will probably be discovered.

Because the boy was with severe and chronic infection was treated using the combination of quinine plus clindamycin for 6 weeks, as recommended by the Infectious Diseases Society of America (IDSA) guidelines (3). Clinical symptoms and parasites may relapse in immunocompromised patients despite 7 to 10 days of antimicrobial therapy and may persist for more than a year if the infection is not adequately treated (18, 19, 21). He recovered completely clinical and did not present any adverse reactions during the 12 months of follow-up.

The report of this human case confirms the existence of the disease and active transmission in Ecuador, as well as its wide geographical distribution, should alert human and veterinary physicians and decision-making authorities of the importance of this emerging zoonosis and the needed research to determine preventive and control strategies. Information on the distribution of *Babesia* species is essential for the diagnosis and prevention of the disease. Unfortunately, surveillance of ticks and tick-borne pathogens do not exist in the country. We recommend strengthening the research capacity in a One Health context in order to develop control strategies that reduce the direct and indirect health and economic burden caused by ticks and tick-borne diseases.

## Data availability statement

The original contributions presented in the study are included in the article/supplementary material, further inquiries can be directed to the corresponding author.

## Ethics statement

Ethical review and approval was not required for the study on human participants in accordance with the local legislation and institutional requirements. Written informed consent to participate in this study was provided by the participants’ legal guardian/next of kin.

## Author contributions

MC: overall coordination, writing, editing, and revision of manuscript. MM-A: diagnosis and management of patient, writing, and editing of manuscript. CB-C, RR-H, and SE: parasitological and molecular diagnosis, writing, and editing of manuscript. PC and DA-R: molecular diagnosis and editing of the manuscript. All authors read and approved the final manuscript.

## Funding

This study was supported by Universidad de las Américas (UDLA), Quito, Ecuador.

## Conflict of interest

The authors declare that the research was conducted in the absence of any commercial or financial relationships that could be construed as a potential conflict of interest.

## Publisher’s note

All claims expressed in this article are solely those of the authors and do not necessarily represent those of their affiliated organizations, or those of the publisher, the editors and the reviewers. Any product that may be evaluated in this article, or claim that may be made by its manufacturer, is not guaranteed or endorsed by the publisher.
